# Improving Data Representation of Metalloproteins in the Protein Data Bank

**DOI:** 10.1063/4.0000858

**Published:** 2025-10-27

**Authors:** Alison Biester, Chenghua Shao, Zukang Feng, Ezra Peisach, Jasmine Y. Young, wwPDB Team, Stephen K. Burley

**Affiliations:** 1 RCSB Protein Data Bank and Institute for Quantitative Biomedicine, Rutgers, The State University of New Jersey, Piscataway, NJ, USA; 2 PDBe, EMBL-European Bioinformatics Institute, Hinxton, Cambridge, United Kingdom; 3 PDBj, Institute for Protein Research, Osaka University, Suita, Osaka, Japan; 4 EMDB, EMBL-European Bioinformatics Institute, Hinxton, Cambridge, United Kingdom; 5 BMRB, UConn Health, University of Connecticut, Farmington, CT, USA; 6 RCSB Protein Data Bank, San Diego Supercomputer Center, University of California San Diego, San Diego, CA, USA; 7 Department of Chemistry and Chemical Biology, Rutgers, The State University of New Jersey, Piscataway, NJ, USA; 8 Rutgers Cancer Institute, Rutgers, The State University of New Jersey, New Brunswick, NJ, USA; 9 Rutgers Artificial Intelligence and Data Science (RAD) Collaboratory, Rutgers, The State University of New Jersey, Piscataway, NJ, USA

## Abstract

The Protein Data Bank (PDB) was established in 1971 as the first open-access digital data resource in biology, initially comprising just seven X- ray crystal structures of proteins. Today, the archive houses more than 225,000 experimentally-determined three-dimensional (3D) structures of biological macromolecules that are freely used by many millions of PDB data consumers worldwide. This wealth of information serves as a cornerstone for research and education endeavors across fundamental biology, biomedicine, biotechnology, and the energy sciences. The Worldwide Protein Data Bank partnership (wwPDB, wwpdb.org) includes five core members (RCSB PDB, PDBe, PDBj, BMRB, and EMDB) and one associate member (PDBc). The wwPDB jointly manages the PDB, EMDB, and BMRB core archives, which adhere to the FAIR (Findability, Accessibility, Interoperability, Reusability) principles emblematic of responsible data stewardship.

Metalloproteins represent an important subset of the archive holdings, constituting more than one-third of PDB structures. Accurate chemical descriptions of metal-containing ligands and their interactions with proteins are valuable to researchers studying these metals in biological processes (e.g., respiration, photosynthesis, enzymatic catalysis). The wwPDB faces challenges in providing consistent chemical descriptions for metal ligands. Oxidation state cannot be determined from 3D structure alone, and coordination geometry can change between the compound initially incubated with a protein and the final protein-bound form of the molecule. However, these are key data for understanding the metal function and reactivity in the protein environment.

To uphold organizational imperatives of consistency and accuracy across the PDB, we are working to address challenges in metal ligand data representation through a “remediation” project spanning the entire archive. Here, we describe our efforts to improve metal ligand accuracy through correcting chemical definitions and to enhance metalloprotein annotation through a new data model. Both the corrections and the enhanced annotation are enabled by adoption of software developed by the metalloprotein research community.

